# The clinical value of the Controlling Nutritional Status score for predicting prognosis in systolic heart failure cases in the vulnerable phase

**DOI:** 10.3389/fnut.2023.1084107

**Published:** 2023-02-07

**Authors:** Jinglin Zhao, Wenli Xie, Suling Ye, Shenglin Zhang, Wenyu Shi, Ming Cui, Lili Wang

**Affiliations:** ^1^Department of Cardiology, The Second Hospital of Dalian Medical University, Dalian, Liaoning, China; ^2^Department of Radiology, The Second Hospital of Dalian Medical University, Dalian, Liaoning, China; ^3^Department of Emergency, The Second Hospital of Dalian Medical University, Dalian, Liaoning, China

**Keywords:** CONUT score, malnutrition, systolic heart failure, vulnerable phase, prognosis

## Abstract

**Background:**

Malnutrition, a commonly encountered complication of heart failure, has an association with poor prognosis. The vulnerable phase of heart failure constitutes the most vulnerable stage of heart failure cases after discharge (usually within 3 months). At present, the prognostic value of Controlling Nutritional Status (CONUT) score in the vulnerable phase of systolic heart failure is unclear.

**Methods:**

Totally 187 systolic heart failure cases were retrospectively assessed at the Second Affiliated Hospital of Dalian Medical University. Based on CONUT score at admission, cases were assigned to 3 groups, including the normal nutrition, and mild and moderate or severe malnutrition groups. The primary endpoint was all-cause death in the 90 days following discharge. The secondary, composite outcome encompassed all-cause death and rehospitalization due to heart failure. Kaplan-Meier method and log-rank test were performed to compare outcome event rates between groups. The independent risk factors for outcome events were obtained by multivariate COX regression analysis. The receiver operating characteristic (ROC) curve analysis and the Delong test were used to compare the prediction performance of the CONUT score and other independent risk factors for all-cause death.

**Results:**

During the 90 days of follow-up, 8.6% of HF patients had the primary endpoint and 23.5% had the secondary outcome. All-cause mortality was markedly elevated in the moderate or severe malnutrition group (Logrank: *p* < 0.001). Compared with the normal nutrition group, composite endpoint events had starkly increased incidence rates in both malnutrition groups, and the incidence increased with the severity of malnutrition (Logrank: *p* < 0.05). Multivariate COX risk analysis revealed higher CONUT score [hazard ratio (HR) = 1.791, 95% confidence interval (CI) 1.379–2.327], age (HR = 1.08, 95% CI 1.028–1.134), B-type natriuretic peptide (BNP) (HR = 1.001, 95% CI 1.000–1.001), and aspartate aminotransferase (AST) (HR = 1.008, 95% CI 1.001–1.015) at admission as independent predictive factors of all-cause mortality. And higher CONUT score (HR = 1.162, 95% CI 1.024–1.318) and lower estimated glomerular filtration rate (eGFR) (HR = 0.98, 95% CI 0.966–0.993) for the secondary endpoint. The addition of the CONUT score significantly increased the predictive performance of age, BNP and AST, as well as their combination for all-cause death (Delong test: all *p* < 0.05).

**Conclusion:**

The CONUT score at admission independently predicts poor prognosis during the vulnerable phase in patients with systolic heart failure and may be combined with conventional risk factors to further improve the predictive efficacy.

## 1. Introduction

Despite significant therapeutic advances achieved in recent years, heart failure (HF) is still considered a global public health concern, with elevated mortality and rehospitalization rate associated with population aging (1). Related researches have reported a peak period of all-cause death and readmission in the early months after discharge (usually within 3 months), known as the vulnerable phase of HF (1). A large registry study published previously, OPTIMIZE-HF, revealed a 15% mortality rate, and a 30% re-hospitalization rate for hospitalized heart failure patients within 3 months after discharge (2). Numerous factors are involved in the pathophysiological events of the vulnerable phase, including a short-term aggravation of hemodynamics due to an inability to alleviate congestion in the index hospitalization, with gradually elevated left ventricular filling pressure (1, 3). Especially in decompensated HF with reduced ejection fraction (HFrEF), given the poor hemodynamic reserve as well as multiple co-morbidities, patient prognosis is relatively poorer in the vulnerable phase (1, 3). In this regard, the post-discharge period is a high-risk period that deserves special attention and close monitoring.

Malnutrition, which decreases the quality of life and prognosis, is prevalent among hospitalized elderly HF cases, especially in advanced disease stages (4). A specific nutritional strategy is still being explored, although not mentioned by any of the existing HF guidelines; while nutritional status assessment is recommended, HF patients would benefit from early nutritional intervention (5, 6). Currently, the assessment of malnutrition risk relies on various nutritional screening tools. The Controlling Nutritional Status (CONUT) score, which is based on serum albumin amounts, lymphocyte content, and total cholesterol amounts, is a comprehensive and objective nutritional screening tool, which can be used to examine protein reserves, calorie depletion, and immune function (7). In patients with HF, CONUT score predicts long-term prognosis (4). The nutritional status of HF cases after discharge may be greatly affected by daily diet care, so the CONUT score might have elevated potential in predicting early prognosis after discharge as a nutritional screening tool. The present work aimed to assess the prognostic value of the CONUT score for systolic HF patients in the vulnerable phase.

## 2. Materials and methods

### 2.1. Study population

**FIGURE 1:**
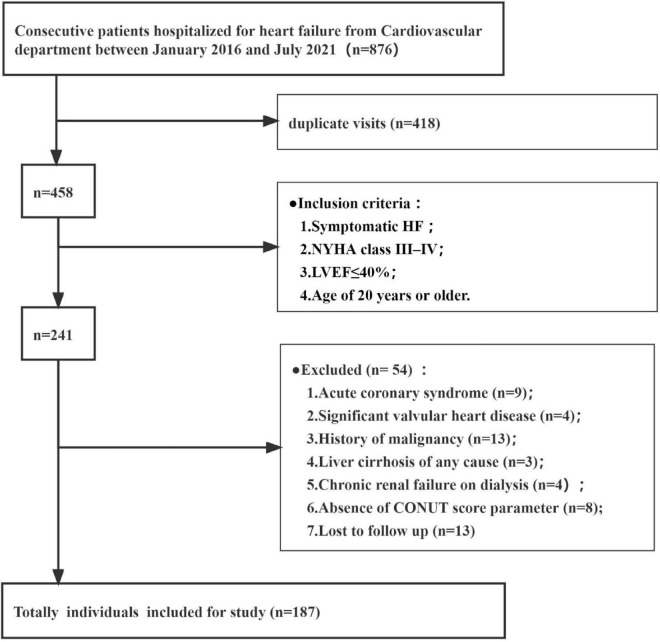
Patient flow chart. CONUT, Controlling Nutritional Status; LVEF, left ventricular ejection fraction; NYHA, New York Heart Association functional classification.

Consecutive patients hospitalized for heart failure in the Second Hospital of Dalian Medical University between January 2016 and July 2021 were retrospectively analyzed. Totally 187 individuals were included according to the following eligibility criteria (Figure 1). The inclusion criteria included symptomatic HF, New York Heart Association (NYHA) class III–IV, left ventricular ejection fraction (LVEF) ≤ 40% on echocardiography and age of 20 years or older. The diagnosis of decompensated HF was based on the European Society of Cardiology (ESC) Heart Failure Guidelines 2021 (5). Exclusion criteria were: significant valvular heart disease (exceeding moderate valvular regurgitation or stenosis), acute coronary syndrome (e.g., myocardial infarction or unstable angina at admission), known history of malignancy, and diagnosed liver cirrhosis of any cause, absence of echocardiographic data, lymphocyte count, total cholesterol levels, serum albumin amounts, and follow-up data. This study did not include patients with chronic renal failure on dialysis.

### 2.2. Baseline data collection

Retrospective data from electronic medical records were collected for assessment, including past medical history, clinical symptoms, medications, and admission laboratory test results, including brain natriuretic peptide (BNP), N-terminal probrain natriuretic peptide (NT-proBNP), uric acid, creatinine, estimated glomerular filtration rate (eGER), cystatin C (Cys-C), urea, alanine aminotransferase (ALT) and aspartate aminotransferase (AST), albumin, total cholesterol (TC), triglyceride, low density lipoprotein (LDL), high density lipoprotein (HDL), hemoglobin, white blood cell (WBC), and lymphocyte absolute amounts. Additionally, echocardiography findings covering left ventricular end-diastolic diameter (LVDD), left atrial diameter (LAD), right ventricular diameter (RVD), and interventricular septal thickness (IVS). Basically, blood samples were collected, and echocardiograms were performed within 72 h of admission. Additionally, body mass index (BMI) was obtained as weight (kg) divided by height (m^2^).

### 2.3. Evaluation of nutritional indexes and groups

The CONUT score evaluates the nutritional status based on laboratory data, i.e., serum albumin (index of protein reserve), total cholesterol (calorie depletion index), and total lymphocyte (indicator of malnutrition-related immune impairment) amounts (7). The patient’s nutritional status was assessed as normal (CONUT scores of 0–1), mild malnutrition (scores of 2–4), moderate malnutrition (scores of 5–8), and severe malnutrition (scores of 9–12; Table 1).

**TABLE 1:** CONUT score.

	Malnutritional state			
Parameter	Normal	Mild	Moderate	Severe
Albumin, g/dL	3.5–4.5	3.0–2.9	2.5–2.9	<2.5
Score	0	2	4	6
Total lymphocyte, × 10^9^/L	>1.6	1.2–1.6	0.8–1.2	<0.8
Score	0	1	2	3
Cholesterol, mg/dL	>180	140–180	100–139	<100
Score	0	1	2	3
Total score	0–1	2–4	5–8	9–12

The HF patients were assigned to three groups, including the normal nutrition (CONUT scores of 0–1), and mild (scores of 2–4), and moderate or severe (5–12) malnutrition groups.

### 2.4. Study endpoint

Within 90 days of discharge during follow-up, all-cause mortality was the primary outcome indicator in this study, and the composite endpoint of all-cause death and rehospitalization for HF was the secondary outcome.

### 2.5. Statistical analysis

Continuous variables with normal distribution are mean ± SD; those with skewed distribution were presented as median and interquartile range. Categorical variables were described by frequency. Multiple group comparisons were conducted by ANOVA followed by *post hoc* Bonferroni’s test (continuous variables with normal distribution), the Kruskal-Wallis test (skewedly distributed data), and the χ^2^ test (categorical data). The event-free survival curves were obtained by the Kaplan-Meier method, and compared by the log-rank test. Cox regression model was utilized in multivariable analysis. Backward stepwise regression analysis was performed, including variates showing *p* < 0.05 in univariable regression analysis and those considered clinically significant for survival outcome in HF. The hazard ratio (HR) and 95% confidence interval (CI) were assessed for each index. In order to assess the predictive values of independent risk factors, areas under the curves (AUCs) for ROC were determined, and the Delong test was performed with the MedCalc software to assess differences in AUCs. The value of each index at the maximum Youden index was taken as the cut-off value. SPSS version 22.0 (IBM, Chicago, USA) was used for all other statistical analyses. *p* < 0.05 were deemed statistically significant.

## 3. Results

### 3.1. Patient characteristics

The baseline clinical features of HFrEF cases classified by CONUT score are listed in Table 2. Among the 187 cases, 43 (23.0%) had CONUT scores ≥ 5, indicating moderate or severe malnutrition. The moderate or severe malnutrition group showed reduced hemoglobin, triglyceride, and low density lipoprotein (LDL) cholesterol, as well as decreased CONUT score indexes (albumin, total cholesterol, and lymphocyte count), and elevated BNP and NT-proBNP amounts. And the rate of spironolactone use was lower compared to the normal nutritional status group. In addition, the proportion of males with mild malnutrition was lower than in the group with normal nutritional status.

**TABLE 2:** The baseline clinical features of patients categorized according to the CONUT score.

Characteristic	Total (*n* = 187)	CONUT score			*P*-value
		Normal	Mild malnutrition	Moderate or severe malnutrition	
		(0–1; *n* = 42)	(2–4; *n* = 102)	(≥5; *n* = 43)	
**Status and vital signs**					
Age (years old)	66.9 ± 16.4	65.7 ± 13.2	65.8 ± 17.4	70.9 ± 16.4	0.193
Male (%)	132 (70.6)	36 (85.7)	63 (61.8)*	33 (76.7)	0.010
BMI (kg/m^2^)	25.5 ± 3.6	25.9 ± 3.6	25.6 ± 3.9	24.9 ± 2.3	0.450
NYHA (III/IV)					0.538
NYHA III (%)	71 (38.0)	19 (45.2)	37 (36.3)	15 (34.9)	
NYHA IV (%)	116 (62.0)	23 (54.8)	65 (63.7)	28 (65.1)	
Systolic blood pressure (mmHg)	135.5 ± 25.7	135.5 ± 24.3	137.6 ± 26.6	130.5 ± 24.6	0.315
Diastolic blood pressure (mmHg)	83.0 ± 17.5	82.2 ± 18.9	85.3 ± 17.4	78.2 ± 15.4	0.077
Heart rate (beats/min)	90.0 ± 21.0	95.0 ± 20.0	91.0 ± 20.0	85.0 ± 21.0	0.109
**Etiology**					
Ischemia (not acute) (%)	73 (39.0)	15 (35.7)	39 (38.2)	19 (44.2)	0.704
Dilated cardiomyopathy (%)	37 (19.8)	9 (21.4)	21 (20.6)	7 (16.3)	0.800
Valvular disease (%)	17 (9.1)	3 (7.1)	9 (8.8)	5 (11.6)	0.769
Hypertension (%)	98 (52.4)	20 (47.6)	55 (53.9)	23 (53.5)	0.779
**Medical history**					
Prior stroke (%)	18 (9.6)	4 (9.5)	7 (6.9)	7 (16.3)	0.242
Diabetes mellitus (%)	60 (32.1)	13 (31.0)	32 (31.4)	15 (34.9)	0.903
Chronic kidney disease (%)	35 (18.7)	4 (9.5)	21 (20.6)	10 (23.3)	0.207
Atrial fibrillation (%)	84 (44.9)	20 (47.6)	45 (44.1)	20 (47.6)	0.923
**Laboratory data**					
BNP (pg/mL)	1763.4 ± 1067.3	1477.3 ± 915.6	1742.6 ± 1106.7	2095.2 ± 1036.9*	0.040
NT-proBNP (pg/mL)	8140.1 ± 7010.7	6041.7 ± 5474.5	8195.9 ± 6871.4	10101.7 ± 8197.1*	0.034
Uric acid (μmol/L)	517.8 ± 157.4	528.6 ± 145.9	520.4 ± 166.8	500.8 ± 147.2	0.702
Creatinine (μmol/L)	108.5 ± 56.6	105.4 ± 40.9	103.0 ± 39.7	124.9 ± 92.2	0.100
EGFR (ml/min/1.73 m^2^)	67.6 ± 19.7	69.9 ± 16.7	68.2 ± 18.9	63.8 ± 24.0	0.338
Cys-C (mg/L)	1.7 ± 0.8	1.6 ± 0.6	1.6 ± 0.6	1.9 ± 1.2	0.109
Urea (mmol/L)	9.3 ± 4.6	8.6 ± 2.8	9.1 ± 4.6	10.5 ± 5.9	0.130
ALT (U/L)	22.3 (13.8–35.3)	19.6 (13.8–30.3)	24.3 (13.6–40)	22.4 (13.8–31.3)	0.453
AST (U/L)	24.0 (18.1–33.9)	20.5 (17.4–30.7)	25.2 (18.4–33.9)	25.2 (18.4–33.9)	0.265
TG (mmol/L)	1.2 ± 0.5	1.5 ± 0.5	1.1 ± 0.5*	0.9 ± 0.5*‡	<0.001
LDL (mmol/L)	2.3 ± 0.9	3.1 ± 0.7	2.2 ± 0.8*	1.7 ± 0.6*‡	<0.001
HDL (mmol/L)	1.0 ± 0.3	1.1 ± 0.3	1.0 ± 0.3	0.9 ± 0.3	0.158
Hemoglobin (g/L)	135.6 ± 23.3	145.7 ± 17.5	137.9 ± 20.4	120.4 ± 27.5*‡	<0.001
WBC (× 10^9^/L)	7.4 ± 2.7	7.7 ± 1.7	7.1 ± 2.4	7.7 ± 3.9	0.279
Lymphocyte count (×10^9^/L)	1.4 ± 0.8	1.8 ± 0.6	1.5 ± 0.8*	0.9 ± 0.4*‡	<0.001
Albumin (g/L)	36.2 ± 4.5	39.2 ± 4.1	36.3 ± 4.1*	32.9 ± 4.0*‡	<0.001
Total cholesterol (mmol/L)	4.0 ± 1.1	5.0 ± 0.8	3.9 ± 1.1*	3.2 ± 0.9*‡	<0.001
**Echocardiographic data**					
LVEF (%)	32.5 ± 6.4	33.9 ± 5.7	31.5 ± 6.6	33.3 ± 6.2	0.063
LVDD (mm)	60.0 ± 8.6	61.0 ± 7.2	60.2 ± 9.5	58.8 ± 7.3	0.485
LAD (mm)	48.3 ± 9.0	48.6 ± 5.9	48.9 ± 9.9	46.6 ± 9.3	0.367
RVD (mm)	25.4 ± 5.4	24.2 ± 3.7	25.7 ± 5.6	25.9 ± 6.1	0.238
IVS (mm)	10.5 ± 2.1	10.8 ± 1.7	10.4 ± 2.2	10.5 ± 2.1	0.692
**Medications**					
Diuretic (%)	181 (96.8)	42 (100.0)	99 (97.1)	40 (93.0)	0.120
β-Blocker (%)	89 (47.6)	21 (50.0)	51 (50.0)	17 (39.5)	0.483
Spironolactone (%)	178 (95.2)	42 (100.0)	98 (96.1)	38 (88.4)*	0.023
Statin drugs (%)	112 (59.9)	29 (69.0)	57 (55.9)	26 (60.5)	0.341
Sacubitril/valsartan (%)	83 (44.4)	21 (50.0)	49 (48.0)	13 (30.2)	0.095
ACE-I/ARB (%)	25 (13.4)	6 (14.3)	13 (12.7)	6 (14.0)	0.962

*p < 0.05 vs. normal nutrition.

‡*p* < 0.05 vs. mild malnutrition.

ACE-I, angiotensin-converting-enzyme inhibitor; ALT, alanine aminotransferase; ARB, angiotensin II receptor blocker; AST, aspartate aminotransferase; BMI, body mass index; BNP, B-type natriuretic peptide; CB, cannabinoid; CONUT, Controlling Nutritional Status; Cys-C, cysteine C; EGER, eastimated glomerularfiltration rate; HDL, high-density lipoprotein; IVS, interventricular septum; LAD, left atrial diameter; LDL, low-density lipoprotein; LVDD, left ventricular diastolic diameter; LVEF, left ventricular ejection fraction; NT-proBNP, N-terminal probrain natriuretic peptide; NYHA, New York Heart Association functional classification; RVD, right ventricular diameter; TG, triglycerides; WBC, white blood cell.

### 3.2. Nutritional status and prognosis

**FIGURE 2:**
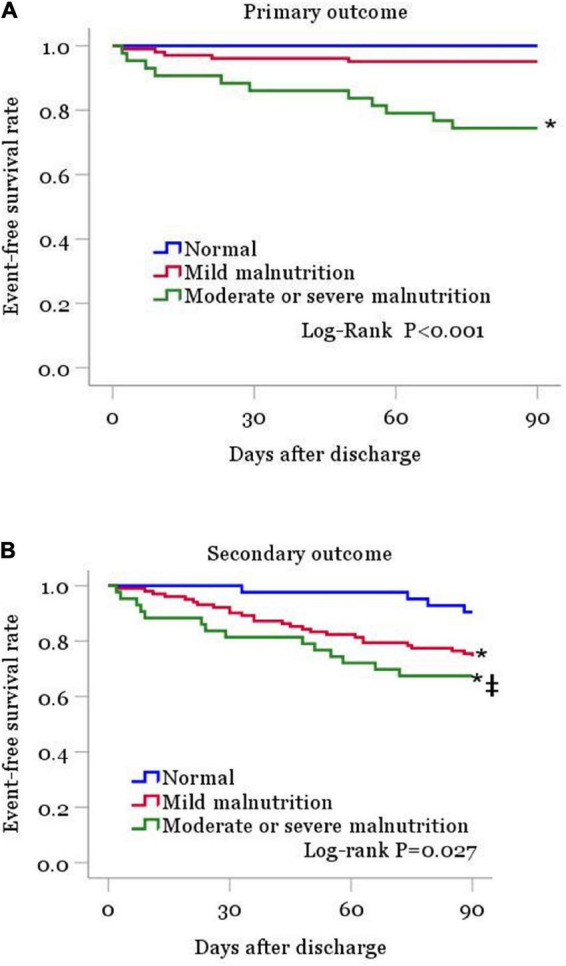
Kaplan-Meier curves are shown for patient groups defined by nutritional status as classified by the CONUT score, for the primary outcome **(A)** and for the secondary outcome **(B)**. CONUT, controlling for nutritional status. *P < 0.05 vs. normal nutrition. ‡*P* < 0.05 vs. mild malnutrition.

Within the 90 days of follow-up among the 187 patients, 16 (8.6%) individuals died of any cause and 28 (15.0%) were readmitted for HF. In a word, 44 (23.5%) patients had a composite endpoint event (all-cause death or rehospitalization for heart failure exacerbation). Kaplan-Meier curve analysis revealed markedly elevated mortality rate in the moderate or severe malnutrition group compared with the normal nutrition group (log-rank test: *p* < 0.001, Figure 2A). All-cause death rates were similar in the normal nutritional status and mildly malnourished groups. For the secondary outcome event, the analysis showed remarkably increased incidence in malnourished cases compared with the normal nutritional status group, and this incidence increased with the degree of malnutrition (log-rank test: *p* < 0.05, Figure 2B).

In multivariate regression analysis of primary outcomes (Table 3), the final results showed that advanced age (HR = 1.08, 95% CI 1.028–1.134, *p* < 0.05), elevated BNP (HR = 1.001, 95% CI 1.000–1.001, *p* < 0.05), elevated AST (HR = 1.008, 95% CI 1.001–1.015, *p* < 0.05), and elevated CONUT score (HR = 1.791, 95% CI 1.379–2.327, *p* < 0.001) were risk factors for all-cause mortality within 90 days after discharge. Meanwhile, in secondary outcomes (Table 4), elevated CONUT score (HR = 1.162, 95% CI 1.024–1.318, *p* < 0.05) and decreased eGFR (HR = 0.980, 95% CI 0.966–0.993, *p* < 0.05) independently predicted the composite endpoint within 90 days after discharge.

**TABLE 3:** Cox proportional hazards analysis for risk of all-cause mortality.

Variables	Univariate			Multivariate		
	Hazard ratio	95% CI	*P*-value	Hazard ratio	95% CI	*P*-value
Age	1.063	1.017–1.110	0.007	1.080	1.028–1.134	0.002
Male	1.466	0.533–4.034	0.459	2.197	0.669–7.213	0.194
SBP	0.996	0.977–1.016	0.697	1.007	0.984–1.030	0.567
Hemoglobin	0.974	0.995–0.992	0.006	0.994	0.966–1.023	0.677
EGFR	0.978	0.957–0.999	0.042	0.991	0.966–1.016	0.467
AST	1.008	1.001–1.015	0.026	1.008	1.001–1.015	0.025
BNP	1.001	1.000–1.001	0.001	1.001	1.000–1.001	0.015
LVEF	1.506	0.964–1.157	0.239	1.035	0.904–1.185	0.621
LVDD	0.958	0.907–1.011	0.118	0.992	0.911–1.080	0.847
RVD	1.054	0.976–1.138	0.177	1.065	0.972–1.166	0176
Spironolactone	3.343	0.759–14.718	0.110	5.009	0.954–26.308	0.057
Sacubitril/valsartan	0.915	0.393–2.834	0.915	0.366	0.114–1.179	0.092
CONUT score	1.561	1.313–1.856	<0.001	1.791	1.379–2.327	<0.001

AST, aspartate aminotransferase; BNP, B-type natriuretic peptide; CONUT, Controlling Nutritional Status; EGFR, estimated glomerular filtration rate; LVEF, left ventricular ejection fraction; LVDD, left ventricular diastolic diameter; RVD, right ventricular diameter; SBP, systolic blood pressure.

**TABLE 4:** Cox proportional hazards analysis for risk of secondary outcome.

Variables	Univariate			Multivariate		
	Hazard ratio	95% CI	*P*-value	Hazard ratio	95% CI	*P*-value
Age	1.022	1.001–1.044	0.038	1.020	0.999–1.042	0.068
Male	1.461	0.790–2.700	0.227	1.628	0.864–3.070	0.132
Heart rate	0.98	0.964–0.996	0.013	0.990	0.973–1.007	0.231
SBP	0.994	0.982–1.006	0.3	1.000	0.985–1.014	0.949
Hemoglobin	0.984	0.972–0.997	0.012	1.002	0.984–1.020	0.862
EGFR	0.979	0.966–0.992	0.002	0.980	0.966–0.993	0.003
BNP	1.000	1.000–1.000	0.110	1.000	1.000–1.000	0.415
LVEF	1.022	0.972–1.073	0.398	1.030	0.974–1.089	0.307
LVDD	1.001	0.967–1.037	0.938	1.021	0.981–1.063	0.310
RVD	1.035	0.981–1.093	0.211	1.039	0.984–1.098	0.170
Spironolactone	2.620	0.937–7.325	0.066	2.675	0.875–8.174	0.084
Sacubitril/valsartan	1.616	0.866–3.014	0.131	1.107	0.568–2.155	0.766
CONUT score	1.196	1.056–1.355	0.005	1.162	1.024–1.318	0.020

CONUT, Controlling Nutritional Status; EGFR, estimated glomerular filtration rate; LVEF, left ventricular ejection fraction; LVDD, left ventricular diastolic diameter; RVD, right ventricular diameter; SBP, systolic blood pressure.

### 3.3. CONUT score’s discriminatory ability in risk assessment

**FIGURE 3:**
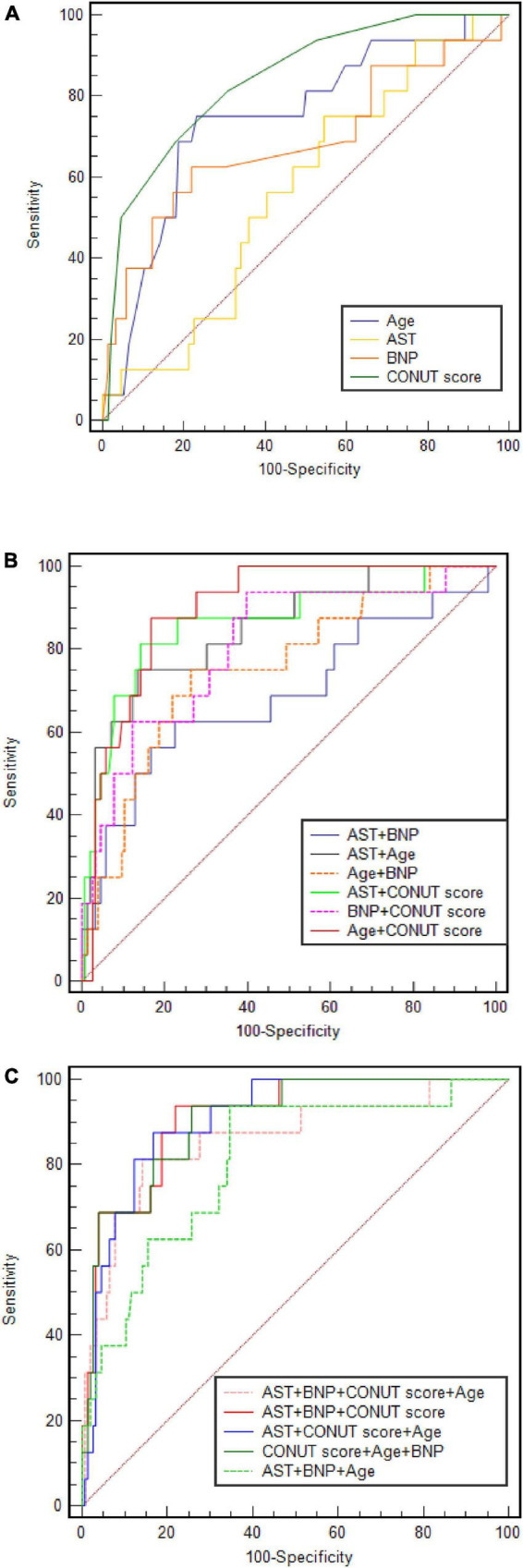
Age, BNP, AST and CONUT score and combined indicators’ ROC curve analysis.

The ROC curves and analysis of discriminatory abilities of Age, BNP, AST, CONUT score and combined indicators for prediction of all-cause death within 90 days of discharge are shown in Figure 3 and Table 5. Among the examined single indicators, the AUC of age (AUC_Age_ 0.734, 95% CI 0.619–0.877), BNP (AUC_BNP_ 0.686, 95% CI 0.524–0.848), and CONUT score (AUC_CONUT score_ 0.841, 95% CI 0.790–0.890) revealed that these factors could significantly predict all-cause death in systolic HF patients after 90 days follow-up. Thereinto, the CONUT score had the highest AUC, albeit with no statistically significant difference compared to age and BNP. At a cut-off value for the CONUT score of 4, sensitivity and specificity in predicting 90-day all-cause death were 68.8 and 80.7%, respectively. However, the AUC of AST was lowest and with none significance (AUC = 0.569, *p* = 0.311). The combine of CONUT score with age (AUC_Age+CONUT score_ 0.889, 95% CI 0.838–0.864), BNP (AUC _BNP+CONUT score_ 0.861, 95% CI 0.800–0.909), or AST (AUC_AST+CONUT score_ 0.843, 95% CI 0.783–0.892) could significantly elevate the AUC compared to AUC_Age_, AUC_BNP_, and AUC_CONUT score_, respectively, (DeLong test: *p* < 0.05). In addition, the combination of the other two factors, except CONUT score, increased AUC to varying degrees compared with the single factor. The combination of age with BNP could significantly elevate the AUC compared to AUC_BNP_ (AUC_Age+BNP_ 0.808, 95% CI 0.741–0.924, DeLong test: *p* < 0.05). Moreover, the combine of CONUT score with either two indicators (AUC_Age+BNP+CONUT score_ 0.905, 95% CI 0.851–0.944; AUC_Age+AST+CONUT score_ 0.896, 95% CI 0.842–0.936; AUC_AST+BNP+CONUT score_ 0.860, 95% CI 0.799–0.900) could significantly elevate the AUC compared to AUC_Age+BNP_, AUC_Age+AST_, and AUC_AST+BNP_, respectively, (DeLong test: *p* < 0.05). Finally, the four-index combination (AUC_Age+AST+BNP+CONUT score_ 0.910, 95% CI 0.857–0.948) can further improve the AUC of any three-index combination. The sensitivity and specificity of the combined four indexes to predict primary outcome were 93.7% and 78.2%, respectively. And the AUC of combine of CONUT score with AST + BNP + Age was significantly different from that of AST + BNP + Age (AUC 0.910 vs. 0.807, DeLong test: *p* < 0.05).

**TABLE 5:** Age, BNP, AST, CONUT score and combined indicators’ ROC curve analysis.

Test outcome variables	AUC	95% CI	Cut-off	Sensitivity%	Specificity%	*P*-value
Age	0.734	0.619–0.877	76	66.4	79.5	0.001
BNP	0.686	0.524–0.848	2026.6	62.5	78.2	0.014
AST	0.569	0.495–0.642	22	75.0	45.3	0.311
CONUT score	0.841^c^	0.790–0.890	4	68.8	80.7	<0.001
Age + CONUT score	0.889^a^	0.838–0.864	0.10	87.5	81.8	<0.001
BNP + CONUT score	0.861^b^	0.800–0.909	0.11	81.3	85.9	<0.001
AST + CONUT score	0.843^c^	0.783–0.892	0.12	75.0	85.9	<0.001
Age + BNP	0.808	0.741–0.924	0.06	93.8	60.3	<0.001
Age + AST	0.740	0.670–0.801	0.09	75.0	80.1	<0.001
AST + BNP	0.688	0.613–0.757	0.08	62.5	77.5	0.024
Age + BNP + CONUT score	0.905^d^	0.851–0.944	0.05	93.8	74.4	<0.001
AST + Age + CONUT score	0.896^e^	0.842–0.936	0.09	87.5	87.1	<0.001
AST + BNP + CONUT score	0.860^f^	0.799–0.900	0.11	81.2	85.9	<0.001
Age + BNP + AST	0.807	0.741–0.864	0.07	93.7	65.3	<0.001
Age + AST + BNP + CONUT score	0.910^g^	0.857–0.948	0.06	93.7	78.2	<0.001

AST, aspartate aminotransferase; AUC, Area under the curve; BNP, B-type natriuretic peptide; CI, confeidence interval; CONUT, Controlling Nutritional Status. Delong test was used to evaluate the differences between AUCs. ^a^*P* < 0.05 vs. Age, ^b^*P* < 0.05 vs. BNP, ^c^*P* < 0.05 vs. AST, ^d^*P* < 0.05 vs. Age + BNP, ^e^*P* < 0.05 vs. Age + AST, ^f^*P* < 0.05 vs. AST + BNP, ^g^*P* < 0.05 vs. Age + AST + BNP.

## 4. Discussion

The vulnerable phase of heart failure refers to the period of high risk of adverse events in the early stage after discharge (usually within 3 months) of HF inpatients (1). Studies have reported that 80–90% of inpatients with heart failure have worsening chronic HF (1). Despite the reduction of hyperemia with diuretics and vasodilators during hospitalization, some patients are still discharged with subclinical hyperemia (1). The short-term hemodynamic deterioration and multiple comorbidities in HF cases after discharge accelerate the occurrence of early adverse events. Corrao et al. performed an analysis of 13,171 cases first hospitalized for heart failure, of whom 4.7 and 4.3%, respectively, died or were re-hospitalized for HF within 30 days after discharge (8). In addition, the EVERST trial, an international multicenter prospective study, followed 4,133 HFrEF cases for an average of 9.9 months. The results showed that 9.6% of HF cases died within 90 days, and 19.4% had rehospitalization events due to cardiovascular disease (9). Consistent with the above study, our follow-up analysis of 187 systolic HF cases demonstrated all-cause death in 8.6% of cases and the composite outcome of all-cause mortality or rehospitalization for HF occurred in 23.5% of patients within 90 days after discharge. The above findings indicate that the vulnerable period in systolic HF is a prominent risk factor for death and rehospitalization due to heart failure, representing an unstable period of the disease. Further investigation is required for assessing the vulnerable period of systolic heart failure.

In recent years, there has been renewed focus on identifying HF cases at high risk of adverse events during the vulnerable phase. In this study, after excluding other important confounding factors affecting the prognosis of heart failure, multivariable regression analysis revealed age, BNP, AST and CONUT score still independently predicted all-cause death within 90 days after discharge in systolic HF cases. Elevated CONUT score and reduced eGFR independently predicted the composite endpoint.

In this study, age was found to independently predict all-cause death in HF cases during the follow-up period. Age represents an important risk factor for cardiovascular disease, and the heart is structurally and functionally altered even without serious injury (10). Aging is typically followed by the thickening and stiffening of the left ventricle, particularly the septum, left atrium enlargement, and cardiac fibrosis (10). Importantly, the reserve function of the heart declines. Studies have found that with increasing age, patients with heart failure have lower hemoglobin levels, worse renal function, higher rates of cardiovascular diseases, and other comorbidities (11). All these factors accelerate the deterioration of the cardiac function, aggravating HF and causing poor prognosis.

B-type natriuretic peptide is crucial in both acute and chronic heart failure. Based on the recommendations of the ESC and the American College of Cardiology Foundation/American Heart Association (ACCF/AHA), BNP is currently regarded as the most reliable biomarker for diagnosing and assessing the severity and prognosis of HF (5, 6). This work revealed baseline BNP independently predicted all-cause death within 90 days upon discharge in HF cases. This is consistent with the EVERST study (9). In addition, short-term follow-up BNP and percent BNP content change are also important predictive variables of all-cause mortality, according to another study that examined heart failure patients for 90 days following discharge (12). Therefore, BNP could help stratify the risk in patients with systolic HF in the vulnerable phase.

Here, we confirmed the independent role of AST in predicting unfavorable outcome in patients with systolic HF. The lower cardiac output in systolic HF leads to hepatic congestion and ischemia, resulting in liver injury. Since AST is mainly found in centrilobular regions far from the dual blood supply to the liver, it is possible that HF patients have high AST (13). In addition, AST is released not only from the liver but also from the myocardium (14). So more severe myocardial injury leads to increased AST values. Previously Lazo et al. also found that elevated AST was significantly associated with troponin and NT-proBNP (15). Meanwhile, some studies have shown that AST/ALT ratio can be used as an independently risk predictor of the severity of systolic heart failure (16). Consistent with our study, a prospective randomized controlled study demonstrated that increased AST values could independently predict mortality within 180 days in patients with HF (17).

There are pathophysiological changes interacting with each other between heart and kidney. In systolic heart failure, due to a significant reduction of cardiac output, a vicious cycle with neurohormonal induction and the renin-angiotensin-aldosterone system as the central link is formed, with strong association with poor outcome in HF. EGFR, the cornerstone of the clinical assessment of renal function, is widely considered a strong predictor of poor outcome in HF cases. Previous reports have shown eGFR significantly increases the risk of death in individuals with HF, both within 30 days and 6 months after discharge (18). This study also found that low eGFR at admission was an important independent predictive factor of composite endpoint events within 90 days upon discharge in systolic HF cases. At present, a number of large randomized controlled trials have proposed improving renal function in systolic HF cases could increase patient survival (19), so renal function in HF cases at admission deserves further attention.

Heart failure is often accompanied by malnutrition. The prevalence of malnutrition in HF patients may range from 5.7 to 78.1% depending on the nutritional assessment tool (20). In this study, the prevalence of malnutrition as assessed by the CONUT score was as high as 77.5% in the systolic heart failure population, which is consistent with previous studies. The CONUT score includes serum albumin, total cholesterol, and total lymphocyte amounts, which can reflect the nutritional metabolism and immune status of the body (7). It is a simple, objective and comprehensive screening tool that covers a variety of factors affecting the prognosis of heart failure. Hyperemia may be the most likely mechanism of malnutrition in systolic HF during the vulnerable phase. Because of intestinal congestion and edema, anorexia and chronic inflammation, insufficient nutrient intake, malabsorption, and elevated resting metabolic rate lead to malnutrition in HF cases (16). In turn, nutrient deficiencies, such as iron deficiency, causing anemia and aggravating cardiac and peripheral dysfunction, increase the risk of death (21). This study also demonstrated hemoglobin amounts were lower in HF cases with moderate or severe malnutrition compared with the normal nutrition and mild malnutrition groups. In addition, malnutrition may result in a deterioration of fluid retention, inflammation, and neurohormone activation (22). For instance, low serum albumin levels cause an imbalance of its function in volume maintenance, and the fluid in blood vessels may transpose to the interstitium, thereby aggravating fluid retention and tissue edema (23). Long-term hypoalbuminemia may lead to myocardial edema, ventricular hypertrophy and reduced myocardial compliance. It is admitted hypoalbuminemia is associated with myocardial fibrosis (23). In addition, the abnormal distribution of blood flow may lead to a decrease of cardiac preload. For cardiovascular diseases with limited ventricular filling, hypotension is more likely to occur and thus tissue and organ perfusion cannot be guaranteed. When the kidney is involved, renin-angiotensin-aldosterone system induction further accelerates HF progression. It is worth noting that in the occurrence and development of heart failure, serum albumin not only decreases in level, but also changes structurally. These changes reduce the anti-inflammatory and anti-oxidative effects of serum albumin, thereby weakening the counteracting effect of serum albumin on cardiovascular damage caused by cytokines. The above effects can accelerate the development of heart failure and worsen prognosis in malnourished HF cases.

In our study, systolic HF cases with moderate or severe malnutrition had starkly lower survival rate within 90 days after discharge than counterparts with normal or mild nutritional status. The results indicate that malnutrition was a risk factor for adverse events during the vulnerable period of systolic HF, and constitutes a complication that needs to be identified and evaluated in time during hospitalization. There may be several reasons for the lack of differences in primary endpoints between the mild malnutrition and normal nutrition groups: a milder degree of malnutrition, a short duration of disease, a short follow-up period after discharge, and improvement in nutritional status during hospitalization or during the vulnerable phase in mild nutrition cases. Nevertheless, it has to be admitted that our sample size is relatively small, would a larger sample size lead to different results? We will analyze this in further studies. This study found that elevated CONUT score independently predicted all-cause mortality and the composite endpoint in systolic HF cases in the vulnerable phase. These findings are consistent with the EFFORT trial, which patients at high risk of malnutrition had an increased risk of death within 180 days (24.7 vs. 38.4%, HR = 1.65, 95% CI 1.21–2.24, *p* = 0.001). In addition, the risk of major cardiovascular events at 30 days was lowered in HF cases by actively controlling fluid overload and providing personalized nutritional support (17.4 vs. 26.9%, odds ratio = 0.50, 95% CI 0.34–0.75, *p* = 0.001) (24). Nutritional intervention improves symptoms, exercise ability and the quality of life in HF cases. Importantly, it could potentially improve many clinical outcomes, including cardiovascular rehospitalization and cardiovascular death in HF cases (25). For systolic HF cases in the vulnerable phase, timing is important in prognosis. Early identification of mild and short-term reversible malnutrition, rapid risk stratification and implementation of appropriate nutritional interventions may help patients have a longer survival and a better quality of life.

This study found that compared to the traditional risk factors age, BNP and AST, CONUT score was no less effective in predicting all-cause mortality in systolic HF within 90 days after discharge (cutoff score, 4), with sensitivity and specificity of 68.8 and 80.7%, respectively. At present, compared with drop an age, BNP and AST, nutritional status in HF cases has not been paid enough attention in clinical practice, but its predictive value for all-cause mortality of systolic HF within 90 days after discharge of heart failure cannot be ignored. It was found an elevated baseline CONUT score is associated with HF severity, and significantly improved predictive value is obtained with the CONUT score added to conventional risk scores for predicting 1-year all-cause death in HF cases administered cardiac therapy in synchrony (26). Therefore, combining the traditional risk factors with the CONUT score may provide further information in clinical risk assessment, to improve the prediction of adverse outcomes in the vulnerable phase of systolic HF.

Notably, we found that using spironolactone was not correlated with the risk of adverse outcomes during the vulnerable period in systolic heart failure cases. Consistent with our study, Lam et al. reported that in elderly cases with systolic HF, spironolactone use was not correlated with all-cause mortality, HF readmission rates, or the incidence of composite endpoints within 30 days after discharge, despite eligibility for spironolactone treatment (27). Indeed, more than one study has indicated that the use of aldosterone receptor antagonists in the real world does not have the same significant amelioration of outcomes as in previous randomized controlled trials, despite the powerful evidence (28–30). This may be strongly tied to the absence of close laboratory follow-up after discharge from the hospital. The salutary role of spironolactone may be diminished by the deterioration of renal function and the high incidence of hyperkalemia due to unsatisfactory monitoring (31). More rigorous laboratory testing may improve the extent of its effectiveness. In further, in clinical practice, enhanced education of patients on treatment adherence may improve the effectiveness of spironolactone. Similarly, the above may partially interpret the potential efficacy-effectiveness dissociation of sakubatril/valsartan from the risk of adverse outcomes in the study results (27). Finally, another explanation that should not be overlooked is the limitation by small sample size and patient selection bias. Therefore, further expansion of sample size and more detailed patient selection are needed for future studies. This may offset some of the baseline differences in our study. Our study found a lower proportion of male in the mildly malnourished group and a lower rate of spironolactone use in the moderately or severely malnourished group compared to the normally malnourished group. And these findings are in sharply contrasting with several prior studies that displayed no differences between nutritional status groups in patients with heart failure (32, 33).

This study suggests that malnutrition is commonly encountered in systolic HF cases and a simple, objective nutritional assessment should be performed on admission using the CONUT score. Early identification and intervention of malnutrition in HF cases may improve patient prognosis in the vulnerable period. Systolic HF cases with moderate or severe malnutrition may require nutritional management in addition to active improvement of hyperemia to ameliorate the clinical outcomes. Therefore, early identification of malnutrition by the CONUT score and appropriate nutritional intervention can benefit patients.

## 5. Limitations

This study had several limitations. First, it was a retrospective trial, and further prospective studies should be conducted to verify the present results. Secondly, the samples size was small, and larger studies are needed. Finally, we did not assess patients’ nutritional status at discharge to obtain information on changes in nutritional status during hospitalization and their prognostic value in patients with heart failure during the vulnerable period.

## 6. Conclusion

In short, in hospitalized systolic HF cases, malnutrition evaluated by the CONUT score could independently predict all-cause mortality and the composite endpoint during the vulnerable phase. Age, BNP and eGFR are closely associated with poor prognosis in heart failure cases in the vulnerable phase. At the same time, CONUT score may be combined with age and BNP to further provide important prognostic information about systolic HF cases during the vulnerable phase, which can help identify systolic HF cases at high risk of death during the vulnerable phase.

## Data availability statement

The raw data supporting the conclusions of this article will be made available by the authors, without undue reservation.

## Ethics statement

The studies involving human participants were reviewed and approved by Ethics Committee of the Second Hospital of Dalian Medical University. The patients/participants provided their written informed consent to participate in this study.

## Author contributions

JZ, WX, and MC contributed to study conception and design. JZ, WX, and WS contributed to data analysis. JZ and WX drafted and edited the manuscript. SY and SZ contributed to the literature search. MC and LW approved the final version of the manuscript. All authors contributed to the article and approved the submitted version.
